# Dengue and Zika Virus 5′ Untranslated Regions Harbor Internal Ribosomal Entry Site Functions

**DOI:** 10.1128/mBio.00459-19

**Published:** 2019-04-09

**Authors:** Yutong Song, JoAnn Mugavero, Charles B. Stauft, Eckard Wimmer

**Affiliations:** a Department of Molecular Genetics and Microbiology, Stony Brook University, Stony Brook, New York, USA; b Codagenix Inc., Farmingdale, New York, USA; Princeton University; Stanford University School of Medicine; Rockefeller University

**Keywords:** Zika virus, cap-dependent translation, cap-independent translation, dengue virus, internal ribosome entry site, IRES

## Abstract

The *Flavivirus* genus of the *Flaviviridae* family encompasses numerous enveloped plus-strand RNA viruses. Dengue virus (DENV), a flavivirus, is the leading cause of serious arthropod-borne disease globally. The genomes of DENV, like the genomes of yellow fever virus (YFV), West Nile fever virus (WNV), or Zika virus (ZIKV), control their translation by a 5′-terminal capping group. Three other genera of *Flaviviridae* are remarkable because their viruses use internal ribosomal entry sites (IRESs) to control translation, and they are not arthropod transmitted. In 2006, E. Harris’ group published work suggesting that DENV RNA does not stringently need a cap for translation. They proposed that instead DENV translation is controlled by an interplay between 5′ and 3′ termini. Here we present evidence that the DENV or ZIKV 5′ untranslated regions (5′-UTRs) alone have IRES competence. This conclusion is based, first, on the observation that uncapped monocistronic mRNAs 5′ terminated with the DENV or ZIKV 5′-UTRs can efficiently direct translation of a reporter gene in BHK and C6/36 cells and second, that either 5′-UTR placed between two reporter genes can efficiently induce expression of the downstream gene in BHK cells but not in C6/36 cells. These experiments followed observations that uncapped DENV/ZIKV genomic transcripts, 5′ terminated with pppAN… or GpppAN…, can initiate infections of mammalian (BHK) or mosquito (C6/36) cells. IRES competence of the 5′-UTRs of DENV/ZIKV raises many open questions regarding the biology and control, as well as the evolution, of insect-borne flaviviruses.

## Abstract (precis)

Members of the genus *Flavivirus* of *Flaviviridae* are important human pathogens of great concern because they cause serious diseases, sometimes death, in human populations living in tropical, subtropical (dengue virus [DENV], Zika virus [ZIKV], and yellow fever virus), or moderate climates (West Nile virus). Flaviviruses are known to control their translation by a cap-dependent mechanism. We have observed, however, that the uncapped genomes of DENV or ZIKV can initiate infection of mammalian and insect cells. We provide evidence that the short 5′ untranslated region (5′-UTR) of DENV or ZIKV genomes can fulfill the function of an internal ribosomal entry site (IRES). This strategy frees these organisms from the cap-dependent mechanism of gene expression at an as yet unknown stage of proliferation. The data raise new questions about the biology and evolution of flaviviruses, possibly leading to new controls of flavivirus disease.

## INTRODUCTION

The 1988 discovery of the “Internal Ribosomal Entry Site (IRES)” in our laboratory and in Sonenberg’s laboratory changed research on translational control in eukaryotic systems (1, 2). At the time, cap-dependent translation in eukaryotic cells had been elevated to a dogma, which was suddenly punctured by an alternative mechanism.

IRESs were originally discovered in picornavirus genomes (that function as mRNA) where they are long, highly structured RNA segments up to 450 nucleotides (nt) long (1, 2). The IRES in the intragenic region (IGR) of the insect pathogen cricket paralysis virus (CrPV), on the other hand, is only 189 nt long (3). IRESs have also been discovered to function in the expression of cellular genes, and again, they are of different structures and sizes (4). These properties make it difficult to identify IRESs by bioinformatics. The observation of a minute viral IRES (96 or 107 nt long) described here is intriguing.

Members of the family *Flaviviridae* comprise a large group of pathogenic viruses, of which many cause severe diseases in millions of humans globally (5). These viruses are enveloped, plus-strand ssRNA viruses with genomes approximately 11 kb in length that are not 3′ polyadenylated (6). They encode a single polypeptide, the polyprotein, consisting of an array of related structural and nonstructural proteins (5).

It is noteworthy that *Flaviviridae* have evolved into two distinct groups with profound differences in their life cycle, especially in the strategy to control translation. Group one comprises member viruses of the genera *Hepacivirus* (hepatitis C virus), *Pestivirus* (bovine viral diarrhea virus), and *Pegivirus* (GB virus). All of them are blood-borne or are transmitted by contact. These viruses control their translation strictly through internal ribosomal entry sites (IRESs) (6). Group two belongs to the genus *Flavivirus* and contains a very large number of species most of which are transmitted by, and can replicate in, insects or acarine species (arboviruses). The best-known human/primate flavivirus is dengue virus (DENV), a health threat to 2.5 billion humans in tropical and subtropical climates. More recently, Zika virus (ZIKV), an agent closely related to DENV, has emerged as a new dangerous flavivirus that cocirculates with DENV in tropical and subtropical climates. As an accepted rule, each member of the genus *Flavivirus* controls its translation by a “cap-dependent” (m^7^GpppANNN…) mechanism (6).

However, a previous study suggested that DENV can initiate protein synthesis by using a cap-independent manner through interaction between its 5′- and 3′-UTRs (7). Experiments have led the authors to conclude that no IRES-like function is involved. Stimulated by results from E. Harris’ laboratory (7), we tested whether our purified, full-length, noncapped transcript RNAs of DENV and ZIKV cDNAs can infect mammalian or mosquito cells by transfection. Surprisingly, the noncapped flavivirus genomes readily infected mammalian (BHK and Vero) or mosquito cells (C6/36) and produced virus in high titers. Translation is the initial step of replication in infections by naked plus-strand RNA genomes. In our case, there are various hypotheses to explain what could lead to viral protein synthesis directed by the uncapped RNA transcripts: translation of uncapped genomes, translation of genomes that were capped in the cytoplasm, or translation controlled by the genomic 5′-UTRs that harbor IRES competence. These possibilities will be discussed later. We opted to focus on the third hypothesis that predicts cap-independent initiation of translation through IRES function of the small 5′-UTRs.

Our experiments followed established strategies: control of translation in monocistronic mRNAs with *Gaussia* luciferase (*Gluc*) as reporter mRNA, followed by experiments in dicistronic mRNA. The dicistronic mRNAs consisted of the firefly luciferase (*Fluc*) gene and the *Gluc* gene, while the DENV or ZIKV 5′-UTRs were introduced between the *Fluc* and *Gluc* genes (1, 2). The results of the experiments reported here allow us to suggest that the short nucleotide sequences of the 5′-UTRs of DENV (96 nt) and ZIKV (107 nt) are competent to serve as very small IRESs directing initiation of translation.

## RESULTS

### The DENV genomic RNAs can be translated cap dependently and cap independently in both mammalian and mosquito cells.

**FIG 1:**
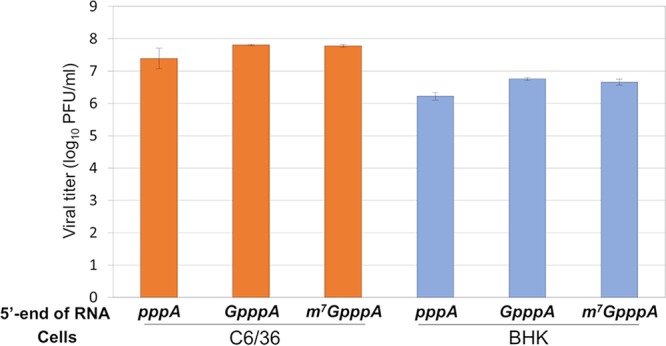
A nonmodified 5′-end DENV genome RNA can produce infectious viral particles in both mammalian and mosquito cells. BHK cells and C6/36 cells seeded on a 12-well plate were transfected with equal amounts of three types of *in vitro* transcripts: (i) a nonmodified 5′pppA-terminated RNA, (ii) an unmethylated 5′ GpppA-terminated RNA, and (iii) a standard m^7^GpppA-capped RNA generated from the synthetic DENV2 infectious clone (DENV2^Syn^ [8]). The cell supernatants were harvested at 5 days posttransfection (CPE can be detected in BHK cells, but not in C6/36 cells). Infectious viral titer (PFU/ml) was determined by plaque assay using BHK cells. The data are expressed as averages from two experiments. Error bars indicate standard deviations.

In all of our experiments described here, we have used our synthetic DENV type 2 clone based on wild-type (wt) DENV2 strain 16681 (8). This plasmid was equipped with a T7 phi2.5 promoter that synthesizes RNA starting with adenosine, the first base (following the cap) of the wt DENV genome (9, 10). Using phage T7 RNA polymerase, we synthesized transcript RNAs and modified the transcripts with one of three different 5′ termini: cap unrelated (pppAN…), a nonfunctional cap (GpppAN…, nonmethylated), or a functional cap (m^7^GpppAN…). Transfection of individual transcripts into mammalian (BHK) cells or into mosquito (C6/36) cells produced virus at the endpoint of experiments roughly equal to that of the transcripts with the functional m^7^G cap (Fig. 1). As we have observed previously (8), infectious virus yields were always slightly higher in C6/36 cells than in BHK cells. This observation suggests that DENV infection is more productive in mosquito cells than in mammalian cells.

Both the m^7^GpppA-capped and unmethylated GpppA-capped DENV transcripts produced similar viral titers on BHK cells after transfection (Fig. 1). Therefore, we carried out a detailed assay of infectivity with the two uncapped pppAN- and GpppAN-DENV genome transcripts wondering if we would find major differences in specific infectivity of the *in vitro* RNAs. Samples were harvested daily (up to day 5) from medium and used for focus-forming assays in Vero cells (see Materials and Methods). The result showed that the pppA-DENV2 genome RNA produced viral titers that were 2 to 3 log_10_ units lower than those obtained with the unmethylated GpppA-5′modified RNAs (see Fig. S1 in the supplemental material). This observation is in accordance with a previous report obtained with transcripts of yellow fever virus cDNA (11). We assume that the viruses harvested at the end of the incubation contain genomes with DENV-specific capping groups. The reason is that the viral replication machinery that was newly assembled in the course of the replication cycle will provide newly synthesized genomes with the 5′-terminal modification (12–14). Experiments to test this hypothesis are currently in progress.

10.1128/mBio.00459-19.1 FIG S1 Infectious assays of different amounts of noncapped pppA- and GpppA-terminated DENV transcript RNAs in mammalian cells. Equal amounts of two types of DENV2 RNA transcripts were transfected onto BHK cell monolayers. Culture media were collected daily (up to day 5), and viral titers were measured by a focus-forming assay in Vero cells. Download FIG S1, TIF file, 0.1 MB. Copyright © 2019 Song et al. 2019 Song et al. This content is distributed under the terms of the Creative Commons Attribution 4.0 International license.

As was mentioned in the introduction, there are different possibilities to explain the infectivity of uncapped DENV transcripts (see Discussion). Below we report our experiments to test the DENV and ZIKV 5′-UTRs for IRES competence.

### Translation of monocistronic mRNAs under the control of DENV 5′-UTR variants in mammalian cells.

**FIG 2:**
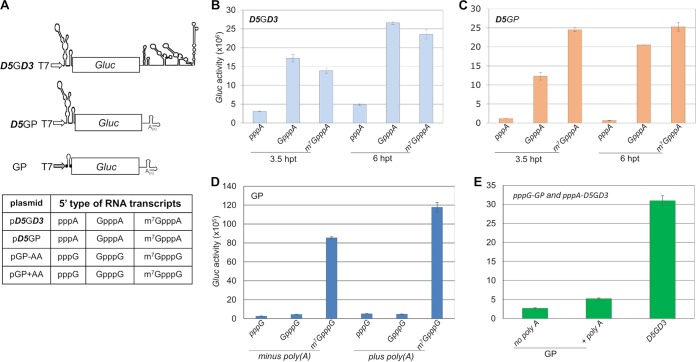
Translation assay directed by the DENV 5′-UTR with different 5′ modifications in BHK cells. (A) Diagrams of DENV monocistronic reporter constructs (details in Materials and Methods). RNA transcripts were generated from a modified/standard T7 promoter. *Gluc*, *Gaussia* luciferase reporter gene. (B to D) Different types of RNA transcripts, ***D5***G***D3*** (B), ***D5***GP (C), and GP (D) RNA transcripts, were generated from a modified T7 promoter (open arrow in panel A for panels B and C) or a standard T7 promoter (solid arrow in panel A for panel D) and then transfected into BHK monolayer cells seeded on a 12-well plate. A certain volume of the medium was harvested for measurement of luciferase activity at different time points (B and C) or at 3.5 h posttransfection (hpt) (D and E). (E) Comparison of *Gluc* expression from noncapped RNA transcripts of ***D5***G***D3*** and GP. The means ± standard errors of the means (SEM) (error bars) from four independent experiments are plotted.

To test whether the uncapped transcripts of the DENV cDNA harbor an activity that allows cap-independent translation, we designed monocistronic mRNAs 5′ terminated with either pppAN, GpppAN, or m^7^GpppAN, followed by the 96-nt-long DENV 5′-UTR sequence (***D5***; Fig. 2) and the *Gluc* ORF (15). We chose two different 3′ termini, the DENV 3′-UTR (***D3***) because of its possible role in DENV translation (16) or the polyadenylated 3′-UTR (P) of poliovirus because of evidence that 3′-terminal poly(A) enhances IRES-mediated initiation of translation (17). Furthermore, the 3′ termini may also contribute to mRNA stability. These constructs are abbreviated as, for example, pppA-***D5***G***D3*** or pppA-***D5***GP, respectively (Fig. 2A). Altogether we prepared 12 different transcripts carrying different termini (detailed in the table in Fig. 2A). Since the DENV 5′ cyclization sequence (5′-CS), located in the 5′ part of core protein-encoding sequences, has not been proven to be necessary for efficient translation (18), we did not retain the 5′-CS in these monocistronic constructs. To standardize the translation experiment, we determined that ∼400 ng of DENV-*Gluc* mRNA (m^7^G-***D5***G***D3***) yielded an optimized signal in transfected mammalian cells (Fig. S2). About 400 ng monocistronic mRNA was therefore selected for all subsequent intracellular translation assays. RNA transcripts were individually transfected into BHK cell monolayers using Lipofectamine 2000, and *Gluc* activities were measured at different time points posttransfection (as indicated; for details, see Materials and Methods). The results showed that the nonmethylated (GpppA) RNAs generated *Gluc* expression levels surpassing the signal of the m^7^G-capped mRNA (Fig. 2B), a result suggesting that in this experiment, the classical m^7^G cap is not required for efficient translation. We expected that the DENV 3′-UTR may contribute to *Gluc* expression particularly with pppA-***D5***G***D3*** mRNA (compare Fig. 2B and C), and, indeed, we observed that the pppA-***D5***G***D3*** reporter RNA produced relatively high *Gluc* activity (pppA-***D5***G***D3***; Fig. 2B) but low *Gluc* with pppG-GP (Fig. 2D). We note that in these experiments, the unrelated 3′ untranslated sequences “minus poly(A)” or “plus poly(A)” were of little, if any, effect on translation of mRNAs without a functional m^7^G cap (GP; Fig. 2D and E). The qRT-PCR results showed that the differences in translational efficiencies are not due to RNA instability of the different reporter RNAs isolated from the transfected cells (Fig. S3).

10.1128/mBio.00459-19.2 FIG S2 Translational efficiency directed by different amounts of ***D5*G*D3*** RNAs in BHK cells. Different amounts of RNA were transfected into BHK monolayer cells seeded on a 12-well plate. The *Gluc* activity was measured at different time points posttransfection as indicated. The means ± SEM from three independent experiments are plotted. Download FIG S2, TIF file, 0.1 MB. Copyright © 2019 Song et al. 2019 Song et al. This content is distributed under the terms of the Creative Commons Attribution 4.0 International license.

10.1128/mBio.00459-19.3 FIG S3 Relative amount of monocistronic RNA transcripts in transfected cells analyzed by qRT-PCR. Total RNA was extracted and isolated by TRIzol reagent from cells transfected with RNA transcripts at different time points posttransfection. RNA levels were measured by qRT-PCR and normalized by GAPDH. The relative amount of m^7^GpppA RNA was artificially set at 1. The means ± SEM from three independent experiments are plotted. *P* values showed no significant difference between samples at indicated time points. Download FIG S3, TIF file, 0.1 MB. Copyright © 2019 Song et al. 2019 Song et al. This content is distributed under the terms of the Creative Commons Attribution 4.0 International license.

These results strongly suggest that the genome of DENV harbors in its 5′-UTR a structure that, independently of a cap structure or the DENV 3′-UTR, is capable of initiating translation.

### In mammalian cells, the DENV 5′-UTR activates internal ribosomal entry in dicistronic mRNAs.

**FIG 3:**
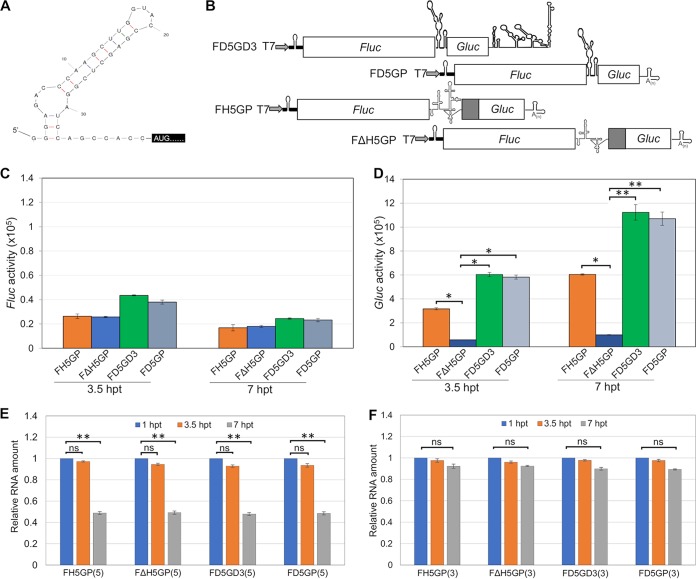
The DENV2 5′-UTR confers IRES activity in dicistronic mRNAs. (A) Sequence and predicted secondary structure of 5′ terminal UTR of all dicistronic constructs in this study are shown. Δ*G* = −13.8 calculated by RNA mfold. (B) Schematic diagrams of dicistronic reporter constructs F*D5*G***D3***, F***D5***GP, F***H5***GP, and F***ΔH5***GP. *Fluc*, firefly luciferase reporter gene. (C) Translation efficiency of 5′ noncapped *Fluc* reporter RNAs. F***H5***GP and F***ΔH5***GP reporter RNAs were included as a positive control and negative control, respectively. (D) *Gluc* expression directed by internal initiation of either the HCV IRES or the DENV2 5′-UTR. *Fluc* and *Gluc* activities were measured at different time points posttransfection (hours posttransfection [hpt]) as indicated. The means ± SEM from three independent experiments are plotted. The *P* values were determined by comparing values to the value for the negative control (F***ΔH5***GP) using two-tailed *t* tests at the indicated time point. Values that are significantly different are indicated by bars and asterisks as follows: *, *P* < 0.05; **, *P* < 0.01. (E and F) Relative amounts of four dicistronic RNA transcripts in transfected cells analyzed by qRT-PCR. Total RNA was extracted and isolated by TRIzol reagent from cells transfected with RNA transcripts at different time points posttransfection. Relative RNA levels were measured by qRT-PCR with either 5′-oligonucleotide primer pairs (E) or 3′-oligonucleotide primer pairs (F) and calibrated by the RNA amount at 1 hpt. The final amount of RNA was normalized to GAPDH. The means ± SEM from three independent experiments are plotted. Values that are significantly different (*P* < 0.01) from the RNA amount at 1 hpt by a two-tailed *t* test are indicated by a bar and two asterisks. Values that are not significantly different are indicated by a bar labeled ns.

On the basis of the results with monocistronic mRNAs, we tested a possible IRES function of the DENV 5′-UTR in dicistronic reporter mRNAs. These mRNAs contained the *Fluc* gene at the 5′ terminus and the *Gluc* gene at the 3′ terminus with the 96 nt of the DENV 5′-UTR placed between the two reporter genes. These mRNAs, designated F***D5***G***D3*** (*Fluc*-DENV2 5′-UTR-*Gluc*-DENV2 3′-UTR) and F***D5***GP (*Fluc*-DENV2 5′-UTR-*Gluc*-PV 3′-UTR), allowed us to assess a possible IRES competence of the 5′-UTR and a contribution of the DENV 3′-UTR to translation initiation of *Gluc* (Fig. 3B). Two additional dicistronic mRNAs, F***H5***GP and F***ΔH5***GP, carry the entire hepatitis C virus (HCV) IRES, or a deletion version thereof (Fig. 3B) and were used as positive and negative controls, respectively. The 5′ termini of these mRNAs (thick lines) are an uncapped, DENV-unrelated sequence (*n* = 41). Importantly, this sequence has the propensity to form a stable hairpin to reduce fortuitous initiation of translation, thereby avoiding an undesired high background level of the *Fluc* signal (Fig. 3A).

Individual transfection of these transcripts into the BHK cells revealed weak upstream *Fluc* expression in all cases but robust downstream *Gluc* expression as controlled by the intercistronic DENV 5′-UTR (Fig. 3). In fact, downstream expression of *Gluc* activated by the DENV 5′-UTR was nearly twice as strong as the activation by the HCV IRES. We note that the nature of the 3′ termini (DENV 3′-UTR [F***D5***G***D3***] or PV 3′-UTR [F***D5***GP]) of the dicistronic mRNAs played at best a minor role in these experiments (Fig. 3D). This result supports our conclusion that the 3′-UTR is unlikely to play a critical role, if any, in upstream *Gluc* expression. Relative *Gluc* expression levels normalized to *Fluc* activity are shown in Fig. S4A. Our qRT-PCR results showed that the difference in translational efficiencies is unrelated to RNA stabilities of the reporter RNAs isolated from the transfected cells (Fig. 3E and F).

10.1128/mBio.00459-19.4 FIG S4 The DENV2 5′-UTR confers IRES activity in dicistronic mRNAs. (A) Relative *Gluc* expression level normalized by *Fluc* activity, associated with Fig. 3. (B) Translation efficiency of 5′ capped *Fluc* reporter RNAs. F***H5***GP and F***ΔH5***GP reporter RNAs were included as positive and negative controls, respectively. (C**)** *Gluc* expression directed by internal initiation of either the HCV IRES or the DENV2 5′-UTR. *Fluc* and *Gluc* activities were measured at different time points posttransfection as indicated. The means ± SEM from four independent experiments are plotted. The *P* values were determined by comparing values to the value for the negative control (F***ΔH5***GP) using a two-tailed *t* test at the indicated time point. **, *P* < 0.01. Download FIG S4, TIF file, 0.1 MB. Copyright © 2019 Song et al. 2019 Song et al. This content is distributed under the terms of the Creative Commons Attribution 4.0 International license.

In addition, the expression of *Fluc* and *Gluc* from the same but capped dicistronic mRNA led to a very high *Fluc* signal (Fig. S4B), as expected. In contrast, the *Gluc* signal (Fig. S4C) from the same capped dicistronic mRNA was very similar to the signal obtained from the corresponding uncapped dicistronic mRNA analyzed earlier (Fig. 3D). This suggests very little influence of ORF1 expression on ORF2 expression under the conditions of our experiment.

We note that the findings reported here do not conform with established translation initiation. We conclude that translation in these dicistronic mRNAs is mediated by an RNA segment with “internal ribosome entry” competence. The DENV2 5′-UTR (96 nt) therefore can function as a minute IRES element that induces robust translation of a downstream gene but only very weak translation of an upstream gene.

### The DENV 3′-UTR does not play a critical role in the noncanonical translation initiation in mammalian cells.

Following the strategy employed for the DENV 5′-UTR, we placed the long 3′-UTR (451 nt) into the intergenic region of the dicistronic mRNAs (F***D3***GP; Fig. S5A) and tested the activity of the reporter genes after transfection into BHK cells. The 3′-UTR, however, did not initiate significantly noncanonical translation of either the upstream *Fluc* gene or the downstream *Gluc* gene (Fig. S5B and S5C). In contrast, the IRES of encephalomyocarditis virus (EMCV), which was used as an intergenic control (F***E***GP), caused very strong downstream activation of the *Gluc* gene (Fig. S5C).

10.1128/mBio.00459-19.5 FIG S5 Role of the DENV2 3′-UTR in translation initiation. (A) Schematic diagrams of dicistronic reporter constructs F***D3***GP and F***E***GP. (B) Translation efficiency of 5′ noncapped reporter RNAs. Dicistronic F***E***GP and F***ΔH5***GP reporter RNAs (shown in Fig. 3B) were also included as positive and negative controls, respectively. (C) *Gluc* expression directed by internal initiation of either the HCV IRES or the DENV2 3′-UTR. *Fluc* and *Gluc* activities were measured at different time points posttransfection as indicated. The means ± SEM from three independent experiments are plotted. The *P* values were determined by comparing values to the value for the negative control (F***ΔH5***GP) using a two-tailed *t* test at the indicated time point. **, *P* < 0.01. Download FIG S5, TIF file, 0.1 MB. Copyright © 2019 Song et al. 2019 Song et al. This content is distributed under the terms of the Creative Commons Attribution 4.0 International license.

The untranslated regions of flavivirus genomes engage in complex interactions with distant upstream genomic sequences, which have been recognized as crucial for viral proliferation (19, 20). Although the DENV 5′-UTR that we have analyzed so far lacks some of the possible binding sites for the 3′-UTR, we constructed dicistronic mRNAs with both 5′- and 3′-UTRs between the two genes in either orientation (F***D53***GP and F***D35***GP). This allowed us to test whether any interaction takes place between the terminal RNA segments that might yield translation cooperativity during this noncanonical translation initiation. The results suggested that there was some stimulation of downstream translation under the control of F***D35***GP RNA, but the activation of *Gluc* was weak.

### Activation of translation by the DENV 5′-UTR in monocistronic mRNAs in mammalian and mosquito cells.

**FIG 4:**
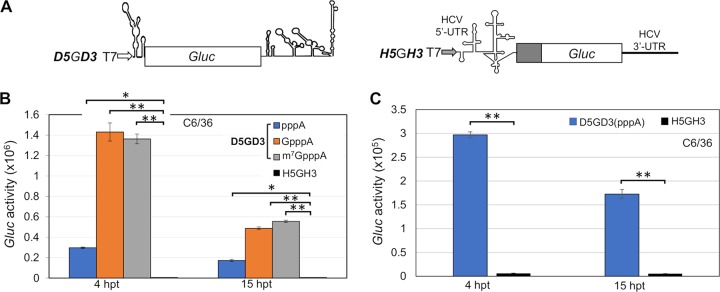
Translation of monocistronic RNAs containing the DENV2 5′-UTR with different 5′ modifications in C6/36 cells. (A) Diagrams of DENV and HCV monocistronic reporter constructs ***D5***G***D3*** and ***H5***G***H3***. (B) Different types of monocistronic ***D5***G***D3*** RNA transcripts and one type of ***H5***G***H3*** reporter RNA were transfected into C6/36 cells, and the *Gluc* activities were measured at different time points posttransfection as indicated. (C) Comparison of *Gluc* expression from only noncapped RNA transcripts of ***D5***G***D3*** and ***H5***G***H3*** at 3.5 hpt. The means ± SEM from three independent experiments are plotted. The *P* values were determined by comparing values to the value for the negative control (***H5***G***H3***) using two-tailed *t* tests at the indicated time point and are shown as follows: *, *P* < 0.05; **, *P* < 0.01.

Being an arbovirus, DENV can infect and replicate in mosquitoes. We therefore expected that in C6/36 mosquito cells the DENV 5′-UTR would initiate cap-independent translation of monocistronic mRNA as described for experiments in BHK cells (Fig. 2). We used ***D5***G***D3*** mRNAs with different 5′ ends (pppA-, GpppA-, and m^7^GpppA-) as described in Fig. 4 and, in addition, monocistronic mRNA with the HCV IRES preceding the *Gluc* gene as a control (***H5***G***H3*;** Fig. 4A). Just as in BHK cells, the noncapped pppA-***D5***G***D3*** RNA produced a low *Gluc* signal in C6/36 cells, an observation that we will address later. mRNAs terminated with 5′ GpppA or m^7^GpppA, however, produced a robust *Gluc* signal (Fig. 4B). In contrast, the ***H5***G***H3*** RNA, containing the HCV IRES upstream of *Gluc* failed to activate translation of *Gluc* in the mosquito cells (Fig. 4C). This supports a recent report that the IRES of a virus of the genus *Hepacivirus* of *Flaviviridae* is nonfunctional in insect cells (21).

### In mosquito cells, the DENV 5′-UTR placed between *Fluc* and *Gluc* in dicistronic mRNA is unable to activate translation of *Gluc*.

Considering the results of monocistronic mRNAs in C6/36 mosquito cells, we expected that the DENV 5′-UTR also would initiate translation of a downstream gene in dicistronic mRNAs in C6/36 mosquito cells. To our surprise, this is not the case.

**FIG 5:**
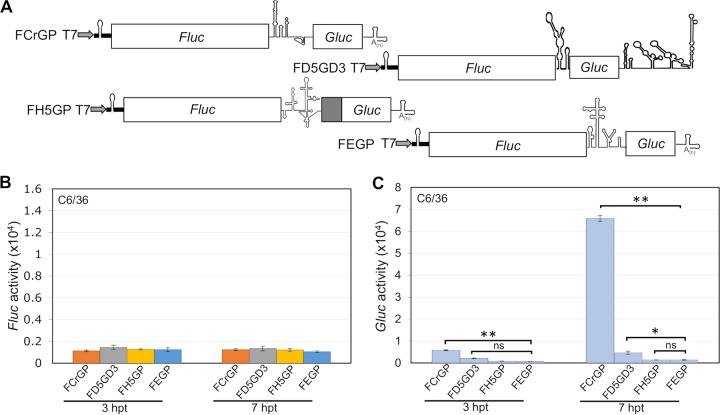
Translation assay of dicistronic constructs containing the DENV 5′-UTR in C6/36 cells. (A) Schematic diagrams of new dicistronic reporter constructs F***E***GP and F***Cr***GP. (B) Translation efficiency of 5′ noncapped dicistronic reporter RNAs. (C) *Gluc* expression directed by internal initiation of either the IGR CrPV IRES, EMCV IRES, HCV IRES, or the DENV2 5′-UTR. *Fluc* and *Gluc* activities were measured at different time points posttransfection as indicated. The means ± SEM from three independent experiments are plotted. The *P* values were determined by comparing values to the value for the negative control (F***H5***GP) using two-tailed *t* test at the indicated time point. Statistical significance is indicated as follows: *, *P* < 0.05; **, *P* < 0.01; ns, not significant.

Specifically, we tested several different noncapped dicistronic mRNAs. These include F***D5***G***D3*** and F***H5***GP, and the new constructs F***E***GP harboring the IRES of encephalomyocarditis virus (EMCV IRES), and F***Cr***GP harboring the small IRES of the intergenic region (IGR) of CrPV, an insect virus, respectively (Fig. 5A). The *Fluc* signals with any of these dicistronic mRNAs were barely detected (Fig. 5B). Unexpectedly, the downstream *Gluc* signals produced by the F***D5***G***D3***, F***H5***GP, and F***E***GP mRNAs were also extremely weak (Fig. 5C). The exception was, not surprisingly, the robust signal induced by F***Cr***GP mRNA, carrying the small IRES of an insect virus (Fig. 5C). Our qRT-PCR results showed that the difference in translational efficiencies is not due to RNA stabilities of the reporter RNAs isolated from the transfected cells (data not shown).

The poor performance of the EMCV and HCV IRESs in mosquito cells is consistent with previous reports (21, 22). It came as surprise, however, that the DENV2 5′-UTR that had shown robust function in monocistronic mRNAs in insect cells was nearly inactive in these cells when placed between the *Fluc* and *Gluc* genes. We speculate that one (or more) specific protein factor(s) may be required in mosquito cells for the minute IRES of the DENV to function and that these factors are either absent or in too low quantities in C6/36 cells to activate translation.

### The 5′-UTR of the ZIKV genome reveals IRES competence similar to that of the DENV 5′-UTR.

**FIG 6:**
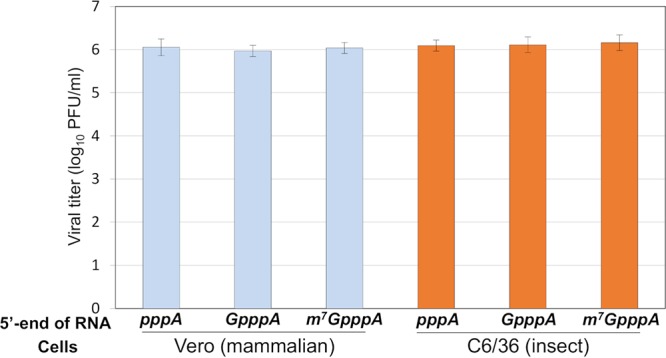
Noncapped ZIKV genome RNAs can produce infectious viral particles in both mammalian and mosquito cells. Vero cells and C6/36 cells seeded on a 12-well plate were transfected with equal amounts of three types of *in vitro* transcripts: (i) a nonmodified 5′pppA-terminated RNA, (ii) a 5′ GpppA-terminated RNA, and (iii) a standard m^7^GpppA-capped RNA generated from the pFLZIKV infectious clone (43). The cell supernatants were harvested at 5 days posttransfection (CPE can be detected in Vero cells, but not in C6/36 cells). Infectious viral titer (PFU/ml) was determined by plaque assay using Vero cells. The data are expressed as averages from two experiments. Error bars indicate standard deviations.

Many features of the molecular biology of ZIKV are closely related to DENV. Since the putative secondary structures of the respective 5′-UTRs share similar folding and stability (Fig. S6), we suspected that the ZIKV 5′-UTR may also have IRES competence. We tested this with experiments very similar to those with the DENV 5′-UTR. Using infectious ZIKV cDNA (strain FSS13025; GenBank accession number KU955593.1), kindly provided by Pei-Yong Shi (Galveston, TX), we first tested the infectivity of full-length ZIKV transcript RNAs, terminated with pppAN, GpppAN, or m^7^GpppAN in mammalian (Vero) and mosquito (C6/36) cells. Vero cells were chosen for ZIKV because plaque assays in these cells yielded superior results compared to those with BHK cells. At the endpoint of the experiments, all uncapped genome variants produced infectious virus in yields roughly equal to that of the genome RNA carrying a functional m^7^G cap (Fig. 6). Possible mechanisms leading to this result will be discussed below in the Discussion.

10.1128/mBio.00459-19.7 FIG S7 Relative *Gluc* expression level normalized by *Fluc* activity (*Gluc*/*Fluc*). The means ± SEM from four independent experiments are plotted. The *P* values were determined by comparing values to the value for the negative control (F***ΔH5***GP) using a two-tailed *t* test at the indicated time point. *, *P* < 0.05; **, *P* < 0.01. Download FIG S7, TIF file, 0.5 MB. Copyright © 2019 Song et al. 2019 Song et al. This content is distributed under the terms of the Creative Commons Attribution 4.0 International license.

**FIG 7:**
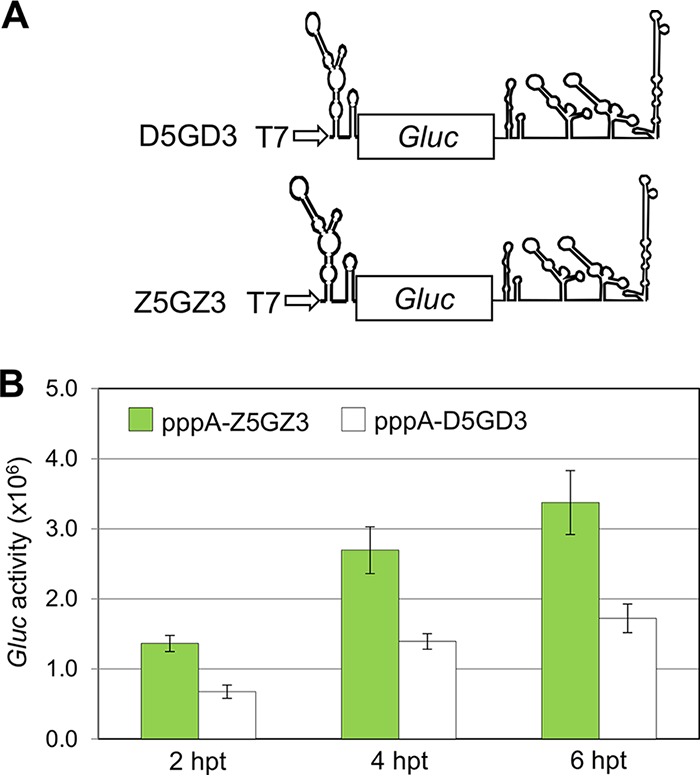
Translation assay of monocistronic constructs containing the ZIKV 5′-UTR in mammalian BHK cells. (A) Schematic diagrams of ***D5***G***D3*** and a new monocistronic reporter construct, ***Z5***G***Z3***. (B) *Gluc* expression level directed by the monocistronic pppA-***D5***G***D3*** and pppA-***Z5***G***Z3*** RNAs. The means ± SEM from four independent experiments are plotted.

**FIG 8:**
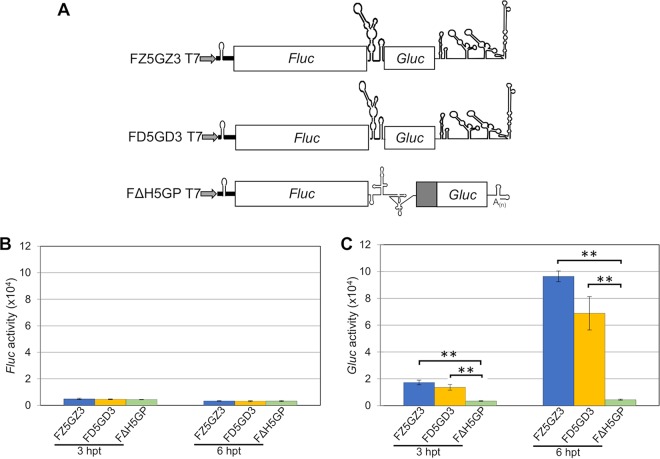
The ZIKV 5′-UTR shows a novel mechanism of translation initiation that is independent of m^7^G cap but dependent on IRES in mammalian BHK cells. (A) Diagrams of F***D5***G***D3***, F***ΔH5***GP, and a new ZIKV dicistronic reporter construct, F***Z5***G***Z3***. (B) Translation efficiency of 5′ noncapped dicistronic reporter RNAs. (C) *Gluc* expression directed by internal initiation of either the HCV IRES or the DENV2 5′-UTR. *Fluc* and *Gluc* activities were measured at different time points posttransfection as indicated. The means ± SEM from four independent experiments are plotted. The *P* values were determined by comparing values to the value for the negative control (F***ΔH5***GP) using a two-tailed *t* test at the indicated time point. **, *P* < 0.01.

We generated monocistronic mRNA ***Z5***G***Z3*** (Fig. 7A) and dicistronic mRNA F***Z5***G***Z3*** (Fig. 8A) and tested these mRNAs in BHK cells. Comparison of ***Z5***G***Z3*** mRNA with ***D5***G***D3*** showed active translation of *Gluc* in both cases. We note that the signal from ***Z5***G***Z3*** surpassed that of the ***D5***G***D3*** RNA (Fig. 7B).

We then tested three dicistronic reporter mRNAs, F***Z5***G***Z3*** (ZIKV 5′- and 3′-UTRs), F***D5***G***D3*** (DENV 5′- and 3′-UTRs), and F***ΔH5***GP (*Fluc*-HCV IRES deletion mutant; Fig. 8A) in BHK cells. Again, these dicistronic RNAs had a noncapped 5′ end (pppA-41 of nonviral nucleotides) with the propensity to form a stable hairpin (Fig. 3A) to avoid high-level background of *Fluc* expression.

Individual transfection of these dicistronic transcripts into the BHK cells revealed that upstream expression of *Fluc* was very weak in all cases (Fig. 8B). Expression of *Gluc*, however, was strong under the control of the intergenic ZIKV or DENV 5′-UTRs (Fig. 8C). Compared with the inactive control RNA F***ΔH5***GP, the *Gluc/Fluc* ratio indicated an approximately 30-fold-higher activity of the F***Z5***G***Z3*** mRNA in the intergenic spacer region (Fig. S7). Our qRT-PCR results showed that the difference in translational efficiencies is not due to RNA instability of the reporter RNAs isolated from the transfected cells (Fig. S8).

10.1128/mBio.00459-19.6 FIG S6 Predicted secondary structure of 5′-UTRs of DENV2 and ZIKV generated by mfold. DENV2 5′-UTR, initial Δ*G* = −28.8 kcal/mol; ZIKV 5′-UTR, initial Δ*G* = −35.90 kcal/mol. Download FIG S6, TIF file, 0.4 MB. Copyright © 2019 Song et al. 2019 Song et al. This content is distributed under the terms of the Creative Commons Attribution 4.0 International license.

10.1128/mBio.00459-19.8 FIG S8 Relative amounts of three dicistronic RNA transcripts in transfected cells analyzed by qRT-PCR. Total RNA was extracted and isolated by TRIzol reagent from cells transfected with RNA transcripts at different time points posttransfection. Relative RNA levels were measured by qRT-PCR with either 5′-end oligonucleotide primers (A) or 3′-end oligonucleotide primers (B) and calibrated by the RNA amount at 1 hpt (used as time 0 posttransfection), respectively. The final amounts of RNAs were normalized by GAPDH. The means ± SEM from three independent experiments are plotted. A significant difference by a two-tailed *t* test compared to control RNA (at 1 hpt) is indicated as follows: **, *P* < 0.01. ns, not significant. Download FIG S8, TIF file, 0.1 MB. Copyright © 2019 Song et al. 2019 Song et al. This content is distributed under the terms of the Creative Commons Attribution 4.0 International license.

### Silencing of the Xrn-1 gene leads to increased expression of reporter genes with our reporter mRNAs.

Throughout the experiments reported here, we observed that our mRNAs, if 5′ terminated with pppAN…, were considerably less active in promoting translation of the reporter gene than mRNA terminated with 5′-GpppAN. We thought that this may be due to the function of Xrn-1, a key component in the major 5′-to-3′ mRNA decay in host cytosols (23). We hypothesized that reducing the function of Xrn-1 (“silencing”) may lead to an increase of the signals of our reporter RNAs, including mRNAs 5′ terminated with pppAN.

**FIG 9:**
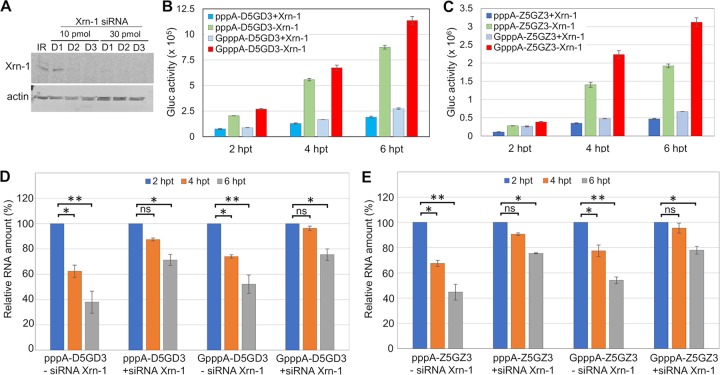
Translation of monocistronic reporter RNAs containing the DENV2/ZIKV 5′-UTRs with different 5′ modifications in A549 cells treated with Xrn-1 siRNA. (A) Xrn-1-specific siRNA was transfected onto A549 cells with control irrelevant siRNA (IR). Total protein was harvested, and Xrn-1 levels were analyzed by immunoblotting assay. Actin served as a loading control. (B and C) A549 cells treated with siRNA (-Xrn-1) and without siRNA (+Xrn-1) were transfected with two types of reporter RNAs, ***D5***G***D3*** (B) and ***Z5***G***Z3*** (C). *Gluc* activities were measured at different time points posttransfection as indicated. Means ± SEM from three independent experiments are plotted. (D and E) RNAs extracted from A549 cells transfected with ***D5***G***D3*** (D) and ***Z5***G***Z3*** (E) at different time points posttransfection were subjected to qRT-PCR analysis generating 5′-end fragments. Each reaction product was finally normalized to GAPDH and presented as the fold change relative to input RNAs. The amount of RNAs at 2 hpt was used as time zero posttransfection and artificially set at 1. The means ± SEM from three independent experiments are plotted. Values that are significantly different from the value for control RNA (at 2 hpt) by a two-tailed *t* test are indicated by bars and asterisks as follows: *, *P* < 0.05; **, *P* < 0.01). Values that are not significantly different (ns) are indicated.

Silencing of the Xrn-1 gene was achieved by transfection of different amounts of Xrn-1-specific siRNAs (Santa Cruz Biotech, TX), together with an irrelevant control siRNA (IR), into A549 cells (derived from human alveolar basal epithelial cells [24]). Transfection of the cells with siRNAs was accomplished by Lipofectamine RNAiMAX (Invitrogen) protocol. Compared to control siRNA (IR)-treated cultures, 10 pmol and 30 pmol of Xrn-1-specific siRNA effectively reduced the expression level of Xrn-1 protein at day 1 and day 2 posttransfection, respectively. In our experiments with reporter mRNAs D5GD3 and Z5GZ3, we used 30 pmol of Xrn-1 siRNA, which prevented the synthesis of Xrn-1 protein after 1 day of treatment (Fig. 9A) (see Materials and Methods).

All ***D5***G***D3*** and ***Z5***G***Z3*** reporter RNAs, irrespective of their 5′ termini (noncapped pppA or GpppA terminated), produced two- to threefold-higher expression levels of *Gluc* in Xrn-1-silenced A549 cells (Fig. 9B and C). This result supports our hypothesis that Xrn-1 silencing may have resulted in the protection of mRNAs and led to increased reporter gene signals. Parallel experiments to test mRNA levels by qRT-PCR seemed to support this conclusion as *Gluc* expression covaried with mRNA stability and stability covaried with the levels of Xrn-1. However, it appeared that GpppA-terminated mRNAs yielded strong *Gluc* signals regardless of the extent of Xrn-1 silencing. We cautiously explain this result with the hypothesis that GpppA termini may protect against 5′-to-3′ degradation. Clearly, this protection would not be available for RNAs terminated with pppAN-. We note that at the early stage of transfection, all mRNAs (pppA or GpppA terminated) were relatively stable, whereas at the wt level of Xrn-1, the relative amount of all reporter mRNAs was reduced very rapidly after 6 h posttransfection (Fig. 9D and E). Among those mRNAs, the noncapped pppA-RNA level decreased the most 6 to 8 h posttransfection (Fig. S9). Similarly, the qRT-PCR derived from the noncapped dicistronic mRNAs F***D5***G***D3*** and F***Z5***G***Z3*** produced almost the same results (data not shown).

10.1128/mBio.00459-19.9 FIG S9 ***D5***G***D3*** (A) and ***Z5***G***Z3*** (B) RNAs, extracted from transfected A549 cells at different time points after transfection, were subjected to qRT-PCR analysis (5′ end target). Each reaction product was finally normalized to GAPDH and presented as the fold change relative to input RNAs. The amount of RNAs at 2 hpt was used as time 0 posttransfection and artificially set at 1. The means ± SEM from three independent experiments are plotted. A significant difference by a two-tailed *t* test compared to control RNA (at 2 hpt) is indicated by asterisks as follows: *, *P* < 0.05; **, *P* < 0.01. ns, not significant. Download FIG S9, TIF file, 0.1 MB. Copyright © 2019 Song et al. 2019 Song et al. This content is distributed under the terms of the Creative Commons Attribution 4.0 International license.

## DISCUSSION

The surprising infectivity of purified uncapped RNA transcripts of DENV or ZIKV cDNA clones in mammalian (BHK and Vero) or insect (C6/36) tissue culture cells (Fig. 1 and 6) can be explained by several hypotheses. These hypotheses include initiation of translation of a small number of intact uncapped viral genomes that allows the formation and amplification of intact replication complexes (25). A second hypothesis draws on reports of cytoplasmic capping of the uncapped RNA molecules posttransfection (26). Cytoplasmic capping occurs predominantly on RNA fragments (not necessarily on mRNA fragments), and the selection process for cytoplasmic capping is poorly understood (26). In Fig. 3C we show that the *Fluc* reporter gene still yields very poor translation after incubation of transcripts in BHK cells for as long as 7 h, a result which may argue against cytoplasmic capping. However, considering the large number of genome transcripts used in the infection (see Fig. S1 in the supplemental material), a rare and fortuitous progression of uncapped transcripts to repaired, capped genomes cannot be excluded.

An alternative hypothesis as to how the noncapped genomes initiated translation and replication is the function of the 5′-UTR as a minute IRES element. The viral polyprotein, once it has been synthesized under the control of the 5′-UTR, will be proteolytically processed and enzyme functions will emerge that not only catalyze genome synthesis but that will also cap the newly synthesized viral RNAs to produce authentic viral genomes. Intracellular repair of modified 5′ termini of viral transcripts has been observed before (27). For example, poliovirus full-length transcripts with the false 5′ end pppGGUUA had a specific infectivity ∼100-fold lower than that of virion RNA. On transfection of pppGGUUA-terminated transcripts into HeLa cells, newly repaired, authentic virion RNAs (VPg-UUA) emerged in the course of replication.

The experiments with uncapped monocistronic mRNAs showed decisively that the DENV 5′-UTR was sufficient to initiate translation of the *Gluc* reporter gene. Moreover, neither the presence of the DENV 3′-UTR nor the presence of a polyadenylated 3′ end was essential for, or did interfere with, *Gluc* synthesis. However, the DENV 3′-UTR at the 3′ end of the ***D5***G***D3*** mRNAs stimulated *Gluc* to some extent (Fig. 2B and C), a result for which we have no explanation.

All monocistronic mRNAs carrying the DENV or ZIKV 5′-UTRs terminated with an uncapped 5′ pppAN- were weak in inducing *Gluc* translation compared with 5′ GpppA-monocistronic mRNAs. We now have evidence that this deficiency is related to RNA stability. In BHK cells, the mRNAs are possibly degraded 5′→3′ by Xrn-1 as shown by silencing the Xrn-1 gene (Fig. 9D and E). Interestingly, mRNAs 5′ terminated with uncapped GpppAN are considerably more resistant to degradation than mRNAs with pppAN- termini regardless of the degree of Xrn-1 silencing (5′-terminal blocking of 5′→3′ exonucleolytic degradation?). We are not sure at present whether other factors play a role in the weak induction of pppAN-terminated mRNAs.

The standard test to detect IRES activity in an RNA sequence is the dicistronic reporter assay (1, 2). Accordingly, we cloned the DENV or ZIKV 5′-UTRs into the intergenic region of dicistronic mRNAs (*Fluc*-x-*Gluc*) followed by transfection into the mammalian BHK cells. We note that the 5′-terminal sequence preceding the *Fluc* ORF in our dicistronic mRNAs was an uncapped sequence capable of forming a stable RNA stem-loop (Fig. 3A). Thus, it is unlikely that this 5′ terminus supports false initiation of translation and high *Fluc* background signals. On the basis of the observed strong signals of the downstream *Gluc* reporter, we conclude that the short 5′-terminal UTRs of DENV or ZIKV are RNAs with IRES competence for internal ribosomal entry. Similar results were obtained with other dicistronic mRNAs that carried either the IRESs of mammalian viruses (HCV or EMCV; ∼350 to ∼450 nt each), or the small intragenic IRES of CrPV (189 nt) (3) in the intergenic regions. We believe that our results with dicistronic mRNA that carry the IRES of HCV, EMCV, or CrPV in the intergenic region, serve as important controls.

Repeating these experiments in C6/36 cells, however, yielded a surprise: neither the DENV 5′-UTR nor the ZIKV 5′-UTR, when placed into the intergenic region of dicistronic mRNAs, expressed IRES function (activation of *Gluc* synthesis) in insect cells. Similarly, when placed into the intergenic region of dicistronic mRNA, the IRESs from other mammalian viruses (HCV and EMCV) were also inactive in C6/36 cells (Fig. 5). On the other hand, a strong *Gluc* signal was obtained when the small intergenic IRES of CrPV (189 nt) was placed between *Fluc* and *Gluc* (Fig. 5C, F***Cr***GP RNA). This experiment supports the validity of the dicistronic mRNA experiment.

The IRESs of HCV, a *Hepacivirus* of *Flaviviridae*, and of EMCV, a cardiovirus, are also inactive in insect cells (Fig. 5) as had been shown by others (21, 22). The reason for the lack of activity of the flavivirus 5′-UTRs in dicistronic mRNA in mosquito cells could point to a lack of specific cellular translation factors, or of ITAFs, or RNA misfolding in insect cells. However, since both DENV and ZIKV are arboviruses, one would have expected an IRES-like function of their 5′-UTRs in insect cells in both mono- and dicistronic mRNAs.

An unorthodox interpretation of these unexpected results is that the IRES competence of DENV or ZIKV 5′-UTRs is a remnant from ancestor viruses of *Flaviviridae* that now belong to the *Hepacivirus, Pestivirus*, and *Pegivirus* genera, e.g., viruses that may never have replicated in arthropods. This could explain the inactivity of the HCV IRES and of the DENV and ZIKV 5′-UTRs when placed into a dicistronic cassette and tested in insect cells.

IRES elements are among the most mysterious RNA structures in eukaryotic molecular biology. As the name suggests (28), an IRES allows initiation of translation of a eukaryotic mRNA independent of the nature of the 5′ terminus of the mRNA. Although IRES elements were discovered 30 years ago, the origin of IRES elements during evolution is unknown. Inexplicably, IRESs are defined by function rather than by size or structure (29). Since single-stranded RNAs can fold innumerable ways to innumerable structures, one can envision that one or more of these structures can lead to complexes between the ribosomal subunits, canonical translation factors, and possibly, cellular proteins (ITAFs). In a large screen, many of those complexes may lead to translation initiation without clear biological relevance. Remarkably, Wellensiek et al. (30) searching for cap-independent translation-enhancing elements, found >12,000 of such entities in the human genome. Most of these elements still await further characterization (30). These studies have been extended recently by Weingarten-Gabbay et al. (31) who reported the existence and function of thousands of human and viral sequences, expressing specific signatures of cap-independent translation activity. We stress that the function of an IRES is expressed most efficiently in cells (or their extracts), which are related to the natural habitat of the virus to which the IRES belongs and thus are likely to provide proteins required for the virus-specific IRES function. For example, translating poliovirus genomes in rabbit reticulocyte lysates revealed an IRES in the C-terminal part of its polyprotein, which turned out to be an artifact (32). Adding protein factors from HeLa cells to the translation extract (33) or translating the poliovirus genome in HeLa extracts altogether (25) completely eliminated this artifact. The cellular proteins necessary for the IRES functions of the DENV and ZIKV 5′-UTRs are not yet known.

Regardless of whether IRESs are large (∼450 nt; poliovirus, encephalomyocarditis virus) or small (e.g., 189 nt; intergenic region IRES in dicistroviruses), they are RNA segments that require the cooperation of cellular protein factors for function. This is in contrast to prokaryotic systems. For example, ORFs in multicistronic mRNAs of Escherichia coli are preceded by the Shine-Dalgarno sequence (S/D sequence (5′-***AGGAGG***-3′) (34, 35) that alone functions as an internal ribosomal entry site by binding to a complementary sequence at the 3′ end of the ribosomal 16S rRNA (5′**-**GATCA***CCUCCU***UA**-**3′). The discovery of eukaryotic IRESs also prompted a search for RNA sequences in the IRES that could function like S/D sequences. Early work concentrated on oligopyrimidine segments in picornavirus IRESs that, however, were found to serve in specific combinations with other segments of the IRES as “starting window” (36, 37). An exceptional case is a 9-nt-long sequence (5′-***CCGGCGGGU***-3′) found in an IRES of the mRNA encoding the *Gtx* homeodomain protein, which activates translation by base pairing to 18S rRNA (38, 39). We have searched in vain in DENV and ZIKV 5′-UTRs for sequences complementary to 18S rRNAs.

Currently, we cannot answer the question why the 5′ UTRs of DENV and ZIKV, presumably of related flaviviruses (West Nile virus [WNV], yellow fever virus [YFV], Japanese encephalitis virus [JEV], etc.), harbor an IRES function. We speculate that a role of the minute flavivirus IRES could emerge during virus infection when cellular mechanisms scale down cap-dependent translation to reduce proliferation of the invading pathogen (40). Moreover, it is possible that flaviviruses continue their translation through the metaphase of cells, when cap-dependent translation is reduced (41).

The work described here raises many questions about flavivirus genetics and control of proliferation. As we have implied above, another intriguing puzzle to be solved is at what stage in evolution one group of the *Flaviviridae* decided to restrict regulation of translation to a specific mechanism at the expense of another mechanism, and why?

## MATERIALS AND METHODS

### Cell cultures.

BHK (baby hamster kidney) cells, A549 cells (adenocarcinomic human alveolar basal epithelial cells), and Vero cells were maintained in Dulbecco’s modified Eagle medium (DMEM) supplemented with 10% fetal bovine serum (FBS) (HyClone Laboratories, Logan, UT), 100 U/ml of penicillin, and 100 µg/ml of streptomycin (Invitrogen). All cells were grown at 37°C in a 5% CO_2_ incubator. Mosquito C6/36 (ATCC, Bethesda, MD), an Aedes albopictus cell line, was cultured in minimal essential medium (MEM) (Gibco BRL) supplemented with 1/100 nonessential amino acids (NEAA) (Gibco BRL), 10% FBS, penicillin G (100 U/ml), and streptomycin (100 mg/ml) at 28°C in a 5% CO_2_ incubator.

### Plasmids. (i) p*D5*G*D3*, pD5GP, pGP, and p*H5*G*H3* monocistronic constructs.

p***D5***G***D3*** or p***D5***GP, containing the entire DENV type 2 5′-UTR sequences (96 nt), *Gluc* encoding sequence (New England Biolabs [NEB]), followed by the DENV2 3′-UTR or unrelated poliovirus 3′-UTR and a poly(A) tail, was constructed by standard cloning strategy. In order to produce robust runoff of *in vitro* transcripts, a modified T7 promoter sequence was placed immediately upstream of the authentic DENV2 5′-UTR starting with an adenosine (10). pGP was modified from a commercially available plasmid pCMV-Gluc (NEB), which contains a set of cellular 5′-noncoding sequences (41 nt) and poliovirus 3′-UTR, followed by a poly(A) tail instead of its original 3′-UTR sequences (Fig. 2A). p***H5***G***H3*** is a monocistronic construct, in which the *Gluc* ORF is flanked by an HCV IRES at the 5′ end and the HCV 3′-UTR at the 3′ end, respectively (Fig. 4).

### (ii) Dicistronic reporter constructs.

All dicistronic reporter constructs, pF***D53***GP, pF***D35***GP, pF***E***GP, pF***D3***GP, pF***D5***G***D3***, pF***Z5***G***Z3***, pF***D5***GP, pF***Cr***GP, pF***H5***GP, and pF***ΔH5***GP plasmids, contain the T7 RNA polymerase promoter linked to a cellular 5′ noncoding sequence, including a stable stem-loop (41 nucleotides [nt]; Fig. 3A) fused to the *Fluc* encoding sequences followed by two termination codons. For pF***H5***GP/pF***ΔH5***GP dicistronic constructs, the second cistron contains an active HCV IRES element or an inactivated HCV IRES element (in which stem-loop III, from nucleotides 133 to 290, within the 5′-UTR is entirely deleted), including the 5′ beginning part of core protein encoding sequences, followed by a cellular ubiquitin sequence, which is then fused with a *Gluc* coding sequence ending with a stop codon. The presence of a cellular ubiquitin sequence is to produce 5′ authentic end of downstream *Gluc* coding sequences. For pF***D53***GP and pF***D35***GP dicistronic constructs, a cap-dependent cellular 5′-UTR was fused to the *Fluc* gene sequence as the first cistron, followed by two stop codons to prevent readthrough. The second cistron consists of sequences of the DENV 5′- and 3′-UTRs in either the 5′→3′ (a Kozak context was inserted between the 3′-UTR and *Gluc* encoding sequence) or 3′→5′ orientation and then directly fused to the *Gluc* reporter gene-encoding sequences and finally an unrelated 3′ noncoding sequence, followed by a poly(A) tail. For pF***D5***G***D3*** and pF***D5***GP dicistronic constructs, translation of the *Gluc* reporter gene is initiated by a DENV 5′-UTR sequence (96 nt) in the presence of DENV 3′-UTR sequence (451 nt) only (F***D5***G***D3***) and an unrelated 3′-noncoding sequence (derived from poliovirus genome sequence), followed by a poly(A) tail (F***D5***GP). For dicistronic constructs pF***E***GP, pF***D3***GP, and F***Cr***GP, the general structure is the same as pF***D5***GP, except that the internal initiation of translation was directed by an EMCV IRES (F***E***GP), a DENV 3′-UTR sequence (F***D3***GP), and CrPV IGR IRES (189 nt, F***Cr***GP), respectively. For the pF***Z5***G***Z3*** construct, translation of the *Gluc* reporter gene is initiated by a ZIKV 5′-UTR sequence (107 nt) in the presence of the ZIKV 3′-UTR sequence (428 nt) at the very 3′ end.

### Flaviviral infectious clones.

The infectious dengue virus serotype 2 clone DENV2^syn^ was synthesized and assembled in our lab (42). An infectious cDNA ZIKV clone (strain FSS13025; GenBank accession number KU955593.1) developed by Pei-Yong Shi’s laboratory at University of Texas Medical Branch (UTMB), Galveston, TX, was kindly given to us (43).

### *In vitro* transcription and RNA transfection in both mammalian and insect cells.

Plasmids were purified by standard Qiagen procedure. The templates for *in vitro* transcription of the reporter RNAs were generated by either PCR (for RNA ending with DENV 3′-UTR) or cleavage linearization [for RNA ending with regular poly(A) tail] to obtain runoff transcripts with the precise 3′ ends, followed by phenol-chloroform extraction and ethanol precipitation. About 1 µg of purified RNA template was transcribed by T7 RNA polymerase (NEB), and the integrity of the RNAs was examined by agarose gel electrophoresis. RNAs were purified by Qiagen RNeasy kit and redissolved in double distilled H_2_O. The concentration of RNA was determined by a Nanodrop-1000 spectrophotometer (Thermo Fisher Scientific). Between 0.4 and 1 µg of transcript RNA was used to transfect BHK or C6/36 cells on a 12-well plate by Lipofectamine 2000 protocol as described previously (44).

Transfected BHK and C6/36 cells were incubated in 5% FBS-DMEM at 37°C (for BHK or Vero cells) and 5% FBS-MEM at 28°C (for C6/36 cells), respectively. BHK or Vero cells were checked daily for cytopathic effect (CPE). When CPE was detected, infection medium was harvested, and fresh 5% FBS-DMEM was added for further incubation until day 9 posttransfection. For transfected C6/36 cells, the medium was harvested at day 5 posttransfection, and fresh 5% FBS-MEM was added for further incubation. The supernatant containing the viral particles was aliquoted and stored at −80°C for further passaging or infection. Viral titers were determined by a modified plaque assay on BHK or Vero cell monolayers using agarose overlay as previously described (45).

### siRNAs and siRNA assay.

Xrn-1 siRNA was purchased from Santa Cruz Biotechnology, Inc., Dallas, TX (catalog no. sc-61811). Another irregular siRNA (si IR) was designed as a duplex with UU 3′ overhangs with the sequence 5′-AAGGACUUCCAGAAGAACAUC-3′ taken from reference 46 and synthesized by Eurofins Genomics LLC (Louisville, KY). Different amounts (10 to 30 pmol) of RNA duplexes were transfected with Lipofectamine RNAiMAX reagent (Thermo Fisher Scientific) onto A549 cells at 60% to 70% confluence. Cells were maintained in standard conditions. At different time points (up to 3 days) posttransfection, cell lysates were prepared and eventually subjected to SDS-PAGE. The silencing effect was examined by Western blotting with specific antibody, anti-Xrn-1 antibody (Boster Biological Technology, Pleasanton, CA).

### RNA isolation from transfected/infected cells and quantitative RT-PCR.

Seventy to 80% confluent BHK or C6/36 cells, seeded on 12-well plate or 35-mm-diameter plate, were transfected with reporter RNAs or infected with virus supernatants at ∼1 PFU per cell. At different time points after transfection (or when CPE was observed during infection of BHK cells), total RNA was extracted from either transfected cells or 200 µl of viral supernatant with 800 µl of TRIzol reagent (Invitrogen), according to the manufacturer’s instructions. Quantitative RT-PCR was performed and analyzed according to the protocols of StepOnePlus Real-Time PCR System (Thermo Fisher Scientific) with 2× SYBR green master mix (Quanta Biosciences). PCR was performed with primers located on the 5′ and 3′ ends of the ORFs of individual reporter genes. Detailed information on oligonucleotide primers is given in Text S1 in the supplemental material. Values were normalized to that of GAPDH, which was amplified with primer pairs of 5′-ATGGCCCCTCCGGGAAACTG and 5′-ACGGAAGGCCATGCCAGTG.

10.1128/mBio.00459-19.10 TEXT S1 Primers for qRT-PCR. Download Text S1, DOCX file, 0.01 MB. Copyright © 2019 Song et al. 2019 Song et al. This content is distributed under the terms of the Creative Commons Attribution 4.0 International license.

### Luciferase activity assay.

At the indicated time points, 10 to 20 µl of the culture medium was taken for the measurement of *Gluc* activity. To assay cell lysates for *Fluc* expression, transfected cells growing in 12-well plates were carefully washed twice with warm phosphate-buffered saline (PBS), followed by treatment with 120 µl of 1× passive lysis buffer (PLB) (Promega) by shaking for 10 to 15 min at room temperature. The supernatant was collected and cleared by centrifugation at 4°C. Luciferase activity was determined in a luminometer (Optocomp I) by the addition of ∼18 µl of sample to 6 to 7 µl of diluted coelenterazine (for *Gluc*; NEB) or ∼20 µl of sample to 15 to 18 µl of *Fluc* substrate (Promega).

### Statistical analysis.

Calculation of the mean and standard deviation (SD) were performed by Microsoft Office 365 (Microsoft Corporation, WA). Statistically significant differences were compared by a two-tailed, unpaired, and unequal variant Student’s *t* test.
