# Supplementary material for: Dengue and Zika Virus 5′ Untranslated Regions Harbor Internal Ribosomal Entry Site Functions
Source: mBio. 2019 Apr 9;10(2):e00459-19. doi: 10.1128/mBio.00459-19 (PMC6456755; doi:10.1128/mBio.00459-19)
Supplement: TEXT S1 [file mBio.00459-19-s0001.docx]

**Supplemental Materials**

**Primers for qRT-PCR:** PCR fragments were amplified in qRT-PCR with the following primers: for 5’-end fragments (129 bps) from mono-cistronic RNAs: 5’-AGTTCTGTTTGCCCTGATCTGC and 5’- AACTTCCCGCGGTCAGCATC; for 5’-end fragments (129 bps) from di-cistronic RNAs: 5’- AAGGCCCGGCGCCATTCTATC and 5’-ATGTTCACCTCGATATGTGCTC; for 3’-end fragments (136 bps) from both mono- and di-cistronic RNAs: 5’- ATCTGTGTGTGGACTGCACAAC and 5’-TTGATCTTGTCCACCTGGCC; for GAPDH fragment (137 bps): 5’- ATGGCCCCTCCGGGAAACTG and 5’- ACGGAAGGCCATGCCAGTG.
